# Supplementary material for: Inhibition of Indoleamine 2,3-Dioxygenase Exerts Antidepressant-like Effects through Distinct Pathways in Prelimbic and Infralimbic Cortices in Rats under Intracerebroventricular Injection with Streptozotocin
Source: Int J Mol Sci. 2024 Jul 8;25(13):7496. doi: 10.3390/ijms25137496 (PMC11242124; doi:10.3390/ijms25137496)
Supplement: Supplementary file 1 [file ijms-25-07496-s001.zip › Supplementary Figure S1.pdf]

## Supplementary Figure S1

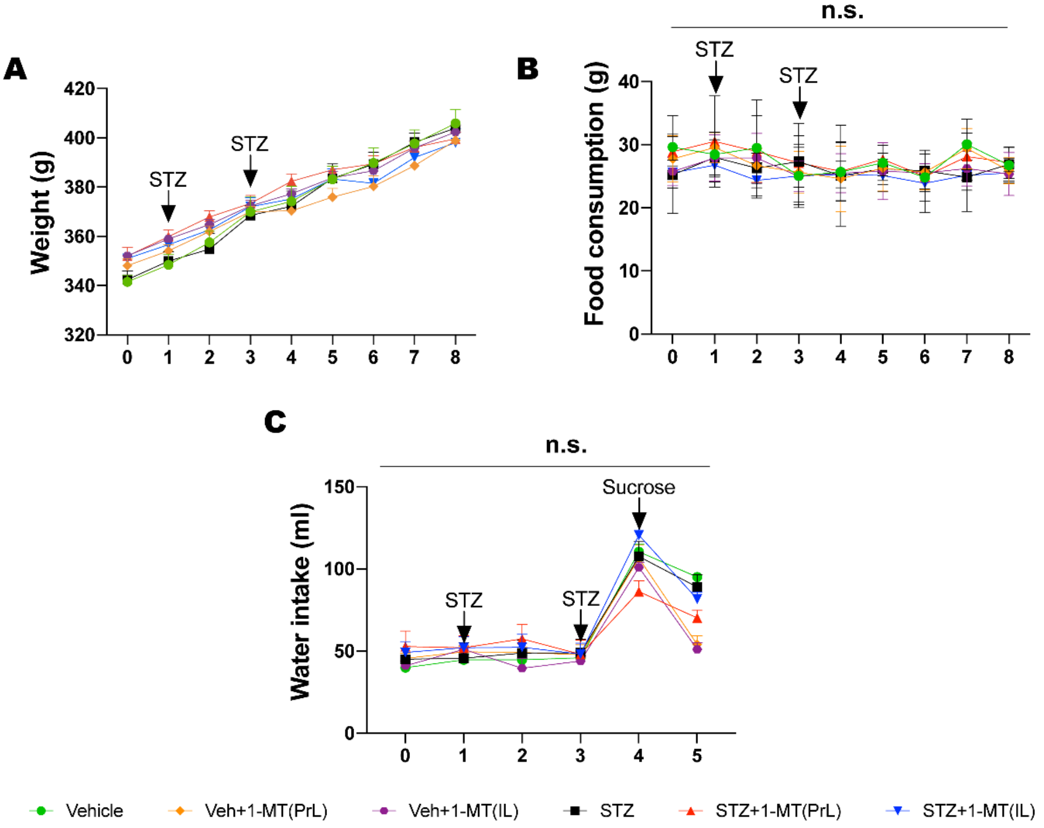


**Supplementary Figure S1.** (A-C) Intra-PrL or -IL administration of 1-MT had no effect on body weight, Food consumption and water intake across the groups (n = 6). The data are expressed as individual values with means ± SEM. One-way ANOVA followed by Tukey's multiple-comparison post hoc test.
