# Supplementary material for: Inhibition of Indoleamine 2,3-Dioxygenase Exerts Antidepressant-like Effects through Distinct Pathways in Prelimbic and Infralimbic Cortices in Rats under Intracerebroventricular Injection with Streptozotocin
Source: Int J Mol Sci. 2024 Jul 8;25(13):7496. doi: 10.3390/ijms25137496 (PMC11242124; doi:10.3390/ijms25137496)
Supplement: Supplementary file 1 [file ijms-25-07496-s001.zip › Supplementary Figure S3.pdf]

Supplementary Figure S3

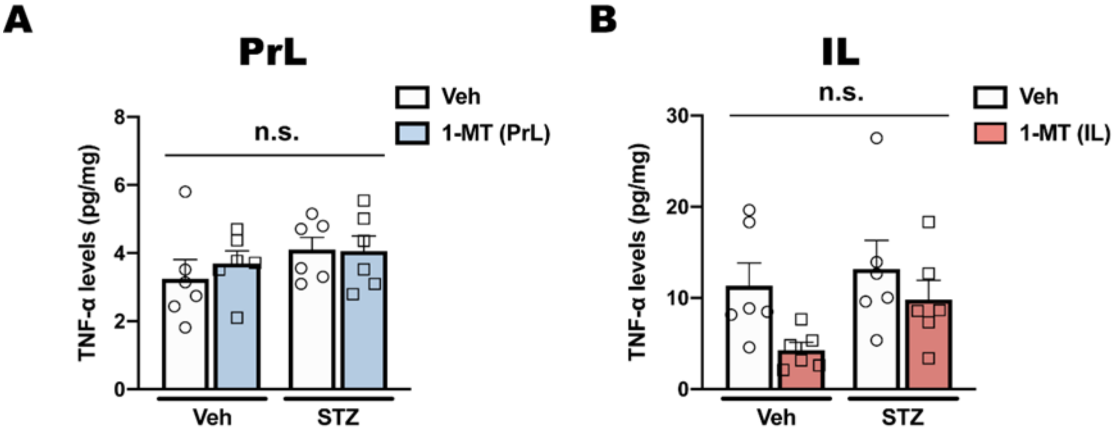

Supplementary Figure S3. (A) Levels of TNF-α in the PrL (n = 6). (B) Levels of TNF-α in the IL (n = 6). The data are expressed as individual values with means ± SEM. Two-way ANOVA followed by Tukey's multiple-comparison post hoc test.
