# Supplementary material for: Inhibition of Indoleamine 2,3-Dioxygenase Exerts Antidepressant-like Effects through Distinct Pathways in Prelimbic and Infralimbic Cortices in Rats under Intracerebroventricular Injection with Streptozotocin
Source: Int J Mol Sci. 2024 Jul 8;25(13):7496. doi: 10.3390/ijms25137496 (PMC11242124; doi:10.3390/ijms25137496)
Supplement: Supplementary file 1 [file ijms-25-07496-s001.zip › Supplementary Table S1.pdf]

Supplementary Table S1

HPLC-MS/MS Parameters

	Mode	Conc. STD (ng/mL)	Parent ion	Daughter ion	Retention time (min)	DP (V)	CE (V)
Trp	Postive	10-10000	205.1	146.1	3.53	40	24
Kyn	Postive	1-1000	209.1	94.1	1.95	60	22
KA	Negative	1-1000	188.0	144.0	3.68	-40	-20
3-HK	Postive	10-10000	225.1	208.1	0.89	45	14
5-HT	Postive	10-10000	192.1	160.1	1.40	35	12
IS	Postive	100	200.0	154.0	3.49	60	20
IS	Negative	100	198.0	181.0	3.47	-60	-20

The linear regression was presented as $y = ax + b$ with a 1/x weight factor. The correlation coefficients for all standard curves showed $r > 0.99$, indicating a good linearity within linear range. The lower limit of quantitation (LLOQ) is defined as signal-noise ratio > 10. The response of the LLOQ was over five times greater than that of the blank sample. The accuracy of LLOQ samples in all matrices was within ±20% (n = 6), and precision was within the acceptance criteria (RSD<15%).

Linearity regression functions of kynurenine metabolites

	Linearity regression functions	r	LLOQ (ng/ml)
Trp	y=0.00057x+0.00369	0.9980	3
Kyn	y=0.0000772x-0.000157	0.9993	0.5
KA	y=0.0127x-0.0106	0.9952	0.5
3-HK	y=0.000865x-0.00136	0.9992	5.5
5-HT	y=0.00139x-0.000961	0.9997	3

Data of accuracy and precision. Compared with the nominal concentrations, the accuracies were ranged from 93.3–112.6 %. The precision values were all less than 15 %. These data indicated that the method provided adequate accuracy and precision.

Accuracy and Precision of kynurenine metabolites

		Normal concentration (ng/ml)	Accuracy (%)	Precision (RSD, %)
	LQC	20	99.4	1.4
Trp	MQC	500	112.6	14.3
	HQC	5000	106.5	3.7
	LQC	2	93.3	13.9
Kyn	MQC	50	99.2	5.9
	HQC	500	98.6	4.0
	LQC	2	103.1	2.9
KA	MQC	50	110.3	13.9
	HQC	500	96.5	10.6
	LQC	20	109.2	5.1
3-HK	MQC	500	104.7	5.1
	HQC	5000	105.3	3.1
	LQC	20	99.2	5.8
5-HT	MQC	500	104.4	3.8
	HQC	5000	101.2	2.4
