# Supplementary material for: Inhibition of Indoleamine 2,3-Dioxygenase Exerts Antidepressant-like Effects through Distinct Pathways in Prelimbic and Infralimbic Cortices in Rats under Intracerebroventricular Injection with Streptozotocin
Source: Int J Mol Sci. 2024 Jul 8;25(13):7496. doi: 10.3390/ijms25137496 (PMC11242124; doi:10.3390/ijms25137496)
Supplement: Supplementary file 1 [file ijms-25-07496-s001.zip › Supplementary Table S2.pdf]

Figure 1

	Con	Veh	STZ-7	STZ-14	STZ-21
A (s)	96.38±7.015	94.25±6.053	170.9±14.27	134.9±7.004	103.9±9.681
B (%)	85.37±3.811	88.43±1.213	57.26±5.961	68.37±4.023	86.47±3.826
C (s)	96.40±6.251	99.83±9.791	54.41±5.386	51.68±4.965	41.71±2.453
D (mm)	23627±2460	23041±1746	23335±2208	22494±1433	23351±1633
F (left)-Familiar	9.642±1.099	13.25±0.8942	11.51±1.403	11.07±1.213	10.84±1.384
F (left)-Novel	20.05±1.067	22.84±1.185	19.85±0.9999	13.95±0.9295	12.77±1.211
F (right)	0.2938±0.02611	0.3138±0.02112	0.2700±0.02104	0.2075±0.01953	0.1938±0.01463

	Veh	STZ
H	1.022±0.01943	1.045±0.09733
I	1.036±0.0236	1.160±0.08819
J	1.033±0.01386	1.086±0.04799
K	1.01±0.02075	0.9491±0.04103

Figure 2

	Veh	STZ
A-ACC	1.743±0.2062	1.765±0.6051
A-PrL	1.572±0.08913	3.265±0.3324
A-IL	1.717±0.1511	2.609±0.2342
A-LHb	0.9228±0.1506	1.029±0.03538
A-DRN	1.247±0.3336	1.364±0.2336
A-Hip	2.636±0.2758	2.755±0.9006
A-LC	1.125±0.2523	1.637±0.1433

	Veh + Veh	Veh + 1-MT	STZ + Veh	STZ + 1-MT
C	1.572±0.08913	0.7338±0.05556	1.082±0.1426	3.265±0.3324
D (mU/mg)	5.539±0.6239	4.967±0.4619	9.225±1.596	5.526±0.5108
E	1.717±0.1511	1.235±0.3886	2.609±0.2342	1.916±0.2777
F (mU/mg)	5.162±0.7217	6.627±0.9636	11.34±2.566	5.515±0.7752

Figure 3

	Veh + Veh	Veh + 1-MT	STZ + Veh	STZ + 1-MT
A (s)	91.83±5.237	87.50±5.353	176.7±7.805	98.83±7.199
B (%)	88.44±2.644	91.63±2.584	59.81±4.157	77.70±4.596
C (s)	140.3±5.346	129.2±10.98	363.7±46.91	119.3±24.72
D (g)	6.000±0.4435	5.550±0.3784	5.600±0.2955	5.800±0.5774
E (s)	92.18±8.540	100.1±7.485	42.47±2.537	61.74±8.205
F (mm)	24382±755.6	25676±802.4	24593±755.6	25301±660.1
G (s)	109.7±2.801	87.50±4.581	159.3±9.912	101.8±5.431
H (%)	89.47±1.615	85.01±1.841	69.34±3.626	81.68±3.543
I (s)	145.7±22.49	151.5±14.79	355.7±26.93	112.0±15.87
J (g)	6.017±0.6483	5.283±0.4600	5.583±0.5218	5.383±0.2868
K (s)	115.3±8.029	108.6±15.96	67.39±9.294	63.65±11.48
L (mm)	22877±1098	23985±726	23169±1685	25373±729.7

Figure 4

	Veh + Veh	Veh + 1-MT	STZ + Veh	STZ + 1-MT
B	282.2±10.65	256.0±5.247	234.3±7.701	282.7±7.851
C	122.8±6.539	143.2±4.854	133.8±4.505	142.2±8.600
E (μm^2)	1965±151.3	1837±126.2	1434±99.62	2211±138.5
F-5 (μm)	6.828±0.3004	6.508±0.3080	3.212±0.1436	4.415±0.1580
F-10 (μm)	6.828±0.3004	6.508±0.3080	4.692±0.1952	5.754±0.2010
F-15 (μm)	10.41±0.2615	9.787±0.2986	6.904±0.2680	9.246±0.3031
F-20 (μm)	11.72±0.4151	11.28±0.4629	8.577±0.3171	11.28±0.4629
F-25 (μm)	9.138±0.4015	10.95±0.3602	7.712±0.2566	9.846±0.3202
F-30 (μm)	7.724±0.3761	8.869±0.2839	6.154±0.2477	8.869±0.2839
F-35 (μm)	7.034±0.2961	8.230±0.2914	5.385±0.2636	8.230±0.2914
F-40 (μm)	3.397±0.2447	4.672±0.1761	4.192±0.2092	5.231±0.1934
G	13.26±0.7698	14.31±0.7398	14.88±0.8373	12.42±0.7142
H (μm^3)	268.3±23.38	301.1±23.51	251.2±17.69	306.5±22.54
J	1.013±0.009485	1.199±0.1431	0.4772±0.05674	1.047±0.1715
K	1.043±0.01537	0.9444±0.07743	0.5505±0.06055	1.007±0.1087
L	1.044±0.01169	1.141±0.1291	0.6735±0.04553	1.143±0.1230
N	1.002±0.01899	2.438±0.3020	1.420±0.1977	2.777±0.7519
O (pg/mg)	27.62±2.702	23.24±3.341	25.16±1.651	23.01±1.980
P (pg/mg)	211.0±11.42	202.6±17.38	252.6±15.05	184.2±23.16

Figure 5

	Veh + Veh	Veh + 1-MT	STZ + Veh	STZ + 1-MT
B	293.5±20.91	287.8±10.37	274.8±14.01	286.8±9.031
C	102.3±10.61	114.8±7.157	143.5±5.566	114.8±3.816
E (μm^2)	1806±122.0	2101±195.1	1832±152.6	1867±158.6
F-5 (μm)	4.585±0.2008	4.359±0.1077	4.789±0.1835	4.359±0.1077
F-10 (μm)	7.231±0.3324	6.453±0.2271	6.702±0.2843	6.453±0.2271
F-15 (μm)	9.969±0.4407	8.906±0.3427	9.596±0.3253	8.759±0.2797
F-20 (μm)	10.57±0.5586	9.625±0.4669	11.26±0.3278	9.776±0.3539
F-25 (μm)	9.938±0.4257	9.750±0.3870	10.44±0.4005	9.086±0.3219
F-30 (μm)	8.292±0.3680	8.516±0.3972	9.421±0.4602	8.879±0.3470
F-35 (μm)	7.246±0.2934	7.625±0.5007	8.123±0.4219	7.655±0.3193
F-40 (μm)	5.877±0.2681	5.703±0.3810	4.860±0.2286	5.724±0.3060
G	12.02±0.9971	14.65±1.089	6.457±0.3550	13.88±1.180
H (μm^3)	290.5±22.38	284.6±22.62	491.3±26.55	284.1±23.78
J	1.045±0.0148	1.899±0.1917	1.515±0.2025	2.164±0.2329
K	1.017±0.0202	0.8558±0.1377	0.6935±0.2552	1.081±0.2173
L	1.055±0.01328	0.9007±0.08878	0.6671±0.161	0.7436±0.2020
N	1.034±0.01337	1.359±0.1742	2.782±0.5891	1.472±0.2453
O (pg/mg)	46.42±8.452	52.03±4.483	90.27±10.36	37.77±5.241
P (pg/mg)	351.6±72.56	282.7±60.79	649.1±94.49	181.7±31.98

Figure 6

	Veh + Veh	Veh + 1-MT	STZ + Veh	STZ + 1-MT
A (ng/g)	25.71±1.603	68.84±14.19	3.633±0.2531	68.39±6.418
B (ng/g)	37.76±1.471	46.51±6.451	43.91±4.286	66.95±7.751
D	1.004±0.01629	1.147±0.07112	0.7384±0.07689	1.111±0.1456
E	1.030±0.009388	0.9807±0.1355	0.9452±0.1287	1.031±0.07748
F (ng/g)	130.0±13.30	62.94±4.850	85.13±6.819	71.77±11.62
G (ng/g)	70.56±5.545	90.93±8.787	106.2±7.499	90.24±12.78
I	1.037±0.01373	1.025±0.0343	0.9141±0.1056	1.037±0.1279
J	1.013±0.004462	1.131±0.03590	1.381±0.1249	1.012±0.1131

Figure 7

	Veh + Veh	Veh + 1-MT	STZ + Veh	STZ + 1-MT
C	6.293±0.2351	6.510±0.2030	3.826±0.1442	6.061±0.1897
D-thin	26.33±1.771	26.94±0.8743	31.50±1.293	26.42±1.350
D-filopodia	20.76±1.181	21.79±0.7996	25.56±1.401	21.51±1.303
D-mushroom	24.09±1.167	23.98±1.050	13.86±0.8821	20.95±1.202
D-stubby	28.82±1.445	27.29±1.131	29.08±1.689	31.13±1.257
F	6.093±0.2262	5.973±0.2069	3.770±0.3090	6.383±0.1954
G-thin	28.73±0.9711	27.42±0.9281	24.76±1.691	27.82±1.218
G-filopodia	24.83±1.152	24.60±1.443	27.93±1.721	24.84±1.241
G-mushroom	20.77±0.9499	20.74±1.195	13.13±1.005	23.39±1.254
G-stubby	25.66±1.047	27.24±0.9723	34.18±1.971	23.95±1.221
I	1.036±0.01012	1.009±0.05011	0.4962±0.09111	1.101±0.07903
K	1.049±0.02050	1.088±0.1066	0.6913±0.04340	10.32±0.06747
