# Supplementary figures and images for: Inhibition of Indoleamine 2,3-Dioxygenase Exerts Antidepressant-like Effects through Distinct Pathways in Prelimbic and Infralimbic Cortices in Rats under Intracerebroventricular Injection with Streptozotocin
Source: Int J Mol Sci. 2024 Jul 8;25(13):7496. doi: 10.3390/ijms25137496 (PMC11242124; doi:10.3390/ijms25137496)

## Supplementary Figure S4

**A**

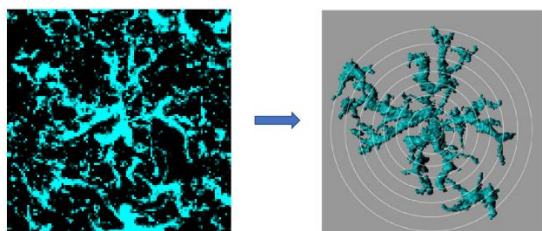

**Supplementary Figure S4.** (A) Schematic diagram of shell analysis.

Supplement: Supplementary file 1 [file ijms-25-07496-s001.zip › Supplementary Figure S4.pdf]

Mode-Positive

5-HT: 5040 ng/ml

3-HK: 5140 ng/ml

Kyn: 506 ng/ml

Trp: 5280 ng/ml

IS: 100 ng/ml

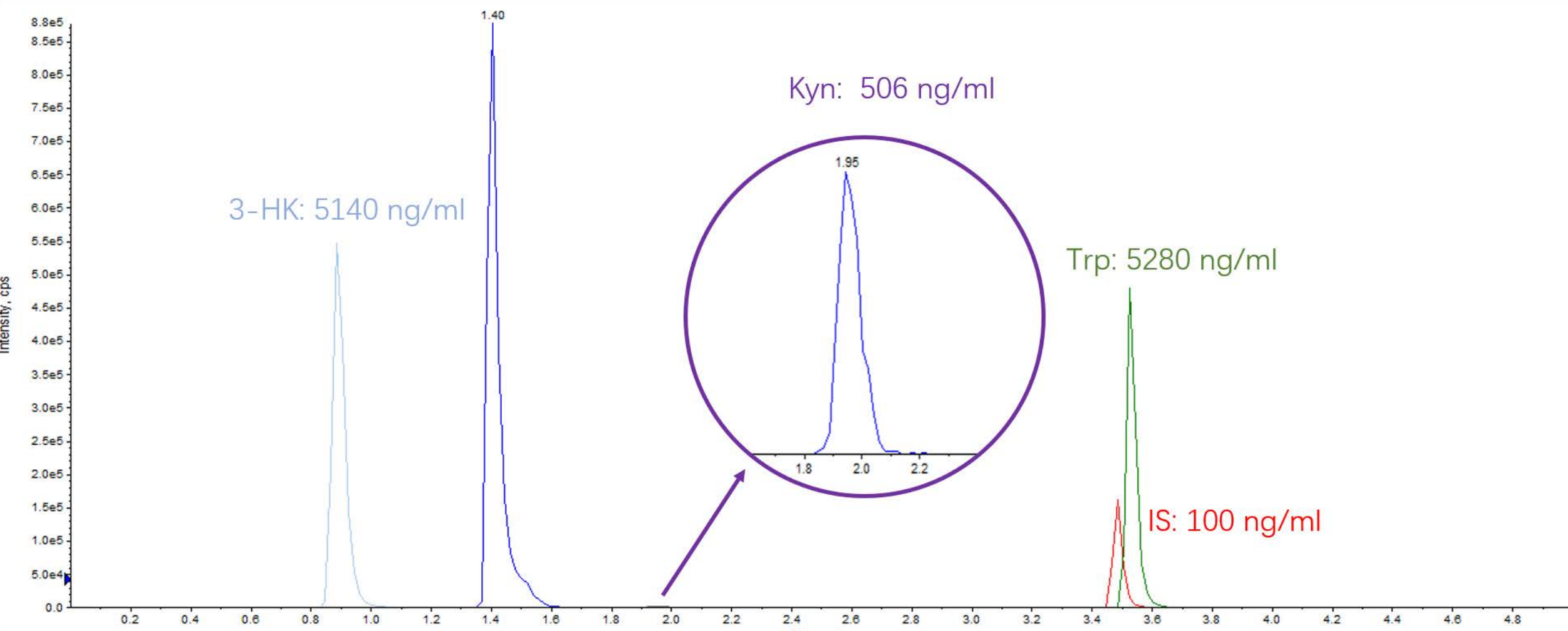

Mode-Negative

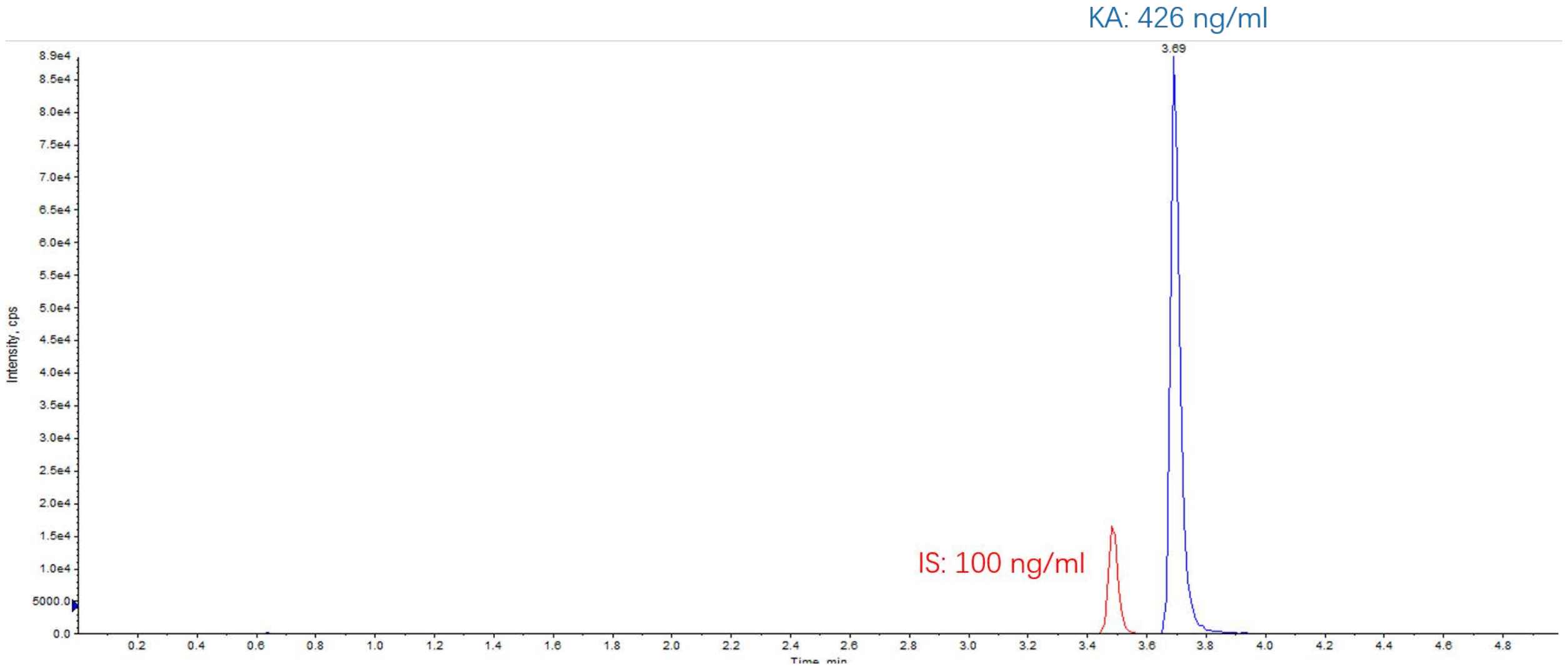

Supplement: Supplementary file 1 [file ijms-25-07496-s001.zip › Supplementary Figure S5.pdf]
